# Supplementary material for: Dynamics of spike-and nucleocapsid specific immunity during long-term follow-up and vaccination of SARS-CoV-2 convalescents
Source: Nat Commun. 2022 Jan 10;13:153. doi: 10.1038/s41467-021-27649-y (PMC8748966; doi:10.1038/s41467-021-27649-y)
Supplement: Supplementary file 1 — Supplementary Information [file 41467_2021_27649_MOESM1_ESM.pdf]

Dynamics of spike- and nucleocapsid specific immunity during long-term follow-up and vaccination of SARS-CoV-2 convalescents

Nina Koerber[1,+], Alina Priller[2,+], Sarah Yazici[2,+], Tanja Bauer[1,3,+], Cho-Chin Cheng[1], Hrvoje Mijočević[1], Hannah Wintersteller[2], Samuel Jeske[1], Emanuel Vogel[1], Martin Feuerherd[1], Kathrin Tinnefeld[1], Christof Winter[4], Jürgen Ruland[4], Markus Gerhard[5], Bernhard Haller[6], Catharina Christa[1], Otto Zelger[7], Hedwig Roggendorf[2], Martin Halle[7], Johanna Erber[8], Paul Lingor[9], Oliver Keppler[10,3], Dietmar Zehn[11], Ulrike Protzer[1,3,+], Percy A. Knolle[2,3,+]

[1]Institute of Virology, Helmholtz-Zentrum München/Technical University of Munich, School of Medicine; [2]Institute of Molecular Immunology and Experimental Oncology, Technical University of Munich, School of Medicine; [3]German Center for Infection Research (DZIF), Munich partner site; [4]Institute of Clinical Chemistry, Technical University of Munich, School of Medicine; [5]Institute of Medical Microbiology, Immunology and Hygiene, Technical University of Munich, School of Medicine; [6]Institute of Medical Informatics, Statistics and Epidemiology, Technical University of Munich, School of Medicine; [7]Institute of Sports Medicine, Technical University of Munich, School of Medicine; [8]Department of Internal Medicine II, Technical University of Munich, School of Medicine; [9]Department of Neurology, Technical University of Munich, School of Medicine; [10]Max von Pettenkofer Institute & Gene Center, Department of Virology, Ludwig-Maximilians-University Munich; [11]Institute of Animal Physiology and Immunology, Technical University of Munich, School of Life Sciences.

Supplementary Figures 1-9

Legends to Supplementary Figures 1-9

Data availability statement

Correspondence:

Percy A. Knolle, MD
Institute of Molecular Immunology and Experimental Oncology
Technical University of Munich, School of Medicine
Ismaningerstr. 22
81675 München
Percy.Knolle@tum.de

Ulrike Protzer, MD
Institute of Virology
Technical University of Munich, School of Medicine/Helmholtz Zentrum München
Trogerstr. 30
81675 München
protzer@tum.de

a

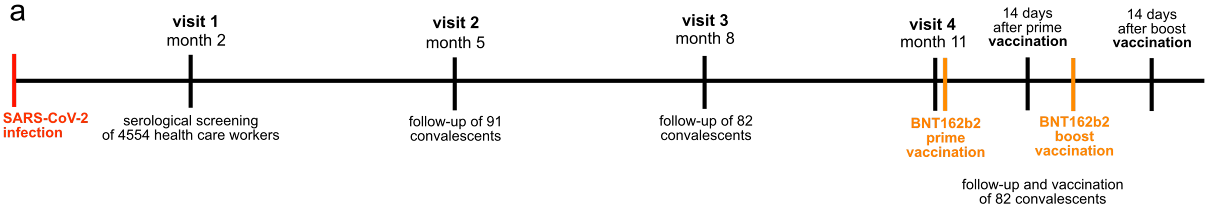

b

study visits	time point	study steps	evaluated parameters	convalescent individuals	naïve individuals
visit 1	month 2	serological screening	anti-SARS-CoV-2 IgG (CLIA, YHLO)	108	4446
		participants with written informed consent to participate in study	naïve individuals: seronegative, paired matching (age, sex, occupation, exposure to COVID-19 patients)	94	53
		confirmation of infection by complementary testing	convalescent: ≥ 2 positive test results for anti-SARS-CoV2 detection by CLIA using assays from Roche, Abbott, Euroimmun, and immunoblots using RecomLine, virus-neutralization activity by cell culture neutralization assay (IC_{50})	91	53
visit 2	month 5	evaluation of SARS-CoV-2 specific immunity	anti-SARS-CoV-2 IgG, virus-neutralization activity by cell culture neutralization assay (IC_{50}), surrogate virus-neutralization activity, (Fluorospot analysis, intracellular cytokine staining when samples available)	91 (88)	53 (52)
visit 3	month 8	evaluation of SARS-CoV-2 specific immunity	anti-SARS-CoV-2 IgG, virus-neutralization activity by cell culture neutralization assay (IC_{50}), surrogate virus-neutralization activity	82	52
visit 4	month 11	evaluation of SARS-CoV-2 specific immunity	anti-SARS-CoV-2 IgG, virus-neutralization activity by cell culture neutralization assay (IC_{50}), surrogate virus-neutralization activity, (Fluorospot analysis when samples available)	82 (49)	53 (39)
vaccination (vacc)	14 days after prime vaccination	evaluation of SARS-CoV-2 specific immunity	anti-SARS-CoV-2 IgG, surrogate virus-neutralization activity, (Fluorospot analysis when samples available)	82 (52)	53 (50)
boost	14 days after boost vaccination	evaluation of SARS-CoV-2 specific immunity	anti-SARS-CoV-2 IgG, surrogate virus-neutralization activity, (Fluorospot analysis, intracellular cytokine staining when samples available)	33 (23)	53 (41)

c

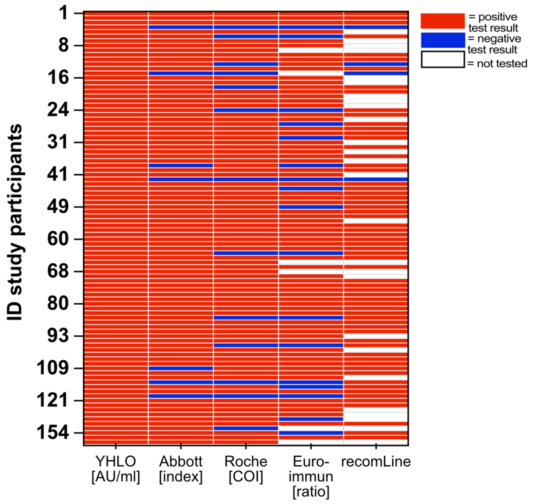

d

	convalescent individuals	naïve individuals
	n = 91	n = 53
**sex**		
male	40 (44.0 %)	20 (37.7 %)
female	51 (56.0 %)	33 (62.3 %)
**mean age ± SD**	39 ± 13 years	42 ± 20 years
**field of work**		
physician	15 (16.5 %)	6 (11.3 %)
nursing	18 (19.8 %)	11 (20.8 %)
medical assistance	5 (5.5 %)	2 (3.8 %)
research / laboratory	10 (11.0 %)	11 (20.8 %)
IT	10 (11.0 %)	5 (9.4 %)
administration	7 (7.7 %)	8 (15.1 %)
student	16 (17.6 %)	7 (13.2 %)
other	10 (11.0 %)	6 (11.3 %)
**patient contact**	49 (53.8 %)	27 (50.9 %)
**COVID-19 patient contact**	16 (17.6 %)	14 (26.4 %)
**risk factors**		
smoking	7 (7.7 %)	11 (20.8 %)
**pre-existing conditions**	19 (20.9 %)	17 (32.1 %)
respiratory conditions	7 (7.7 %)	3 (5.7 %)
cardiovascular conditions	4 (4.4%)	4 (7.5 %)
diabetes mellitus	5 (5.5 %)	0 (0.0 %)
immune deficiency	0 (0.0 %)	1 (1.9 %)
immune suppression	0 (0.0 %)	2 (3.8 %)
other	4 (4.4%)	8 (15.1 %)
**symptoms**	71 (78.0 %)	18 (34.0 %)
fever	49 (53.8 %)	5 (9.4 %)
coughing	46 (50.5 %)	6 (11.3 %)
dyspnea	21 (23.1%)	4 (7.5 %)
cold symptoms	67 (73.6 %)	14 (26.4 %)
loss of smell / taste	45 (49.4 %)	4 (7.5 %)
fatigue / exhaustion	62 (68.1 %)	13 (24.5 %)
headache	45 (49.4 %)	8 (15.1 %)
diarrhea	19 (20.9 %)	3 (5.7 %)

Supplementary Figure 1 | Characteristics of anti-SARS-CoV-2 IgG seropositive study participants

(a) time axis of long-term follow-up in SARS-CoV-2 convalescents. (b) overview of the prospective study. (c) individual characteristics of study participants with ≥ two positive anti-SARS-CoV-2 IgG assays and reports on symptoms during SARS-CoV-2 infection. (d) characteristics of the study cohort for the seroprevalence testing.

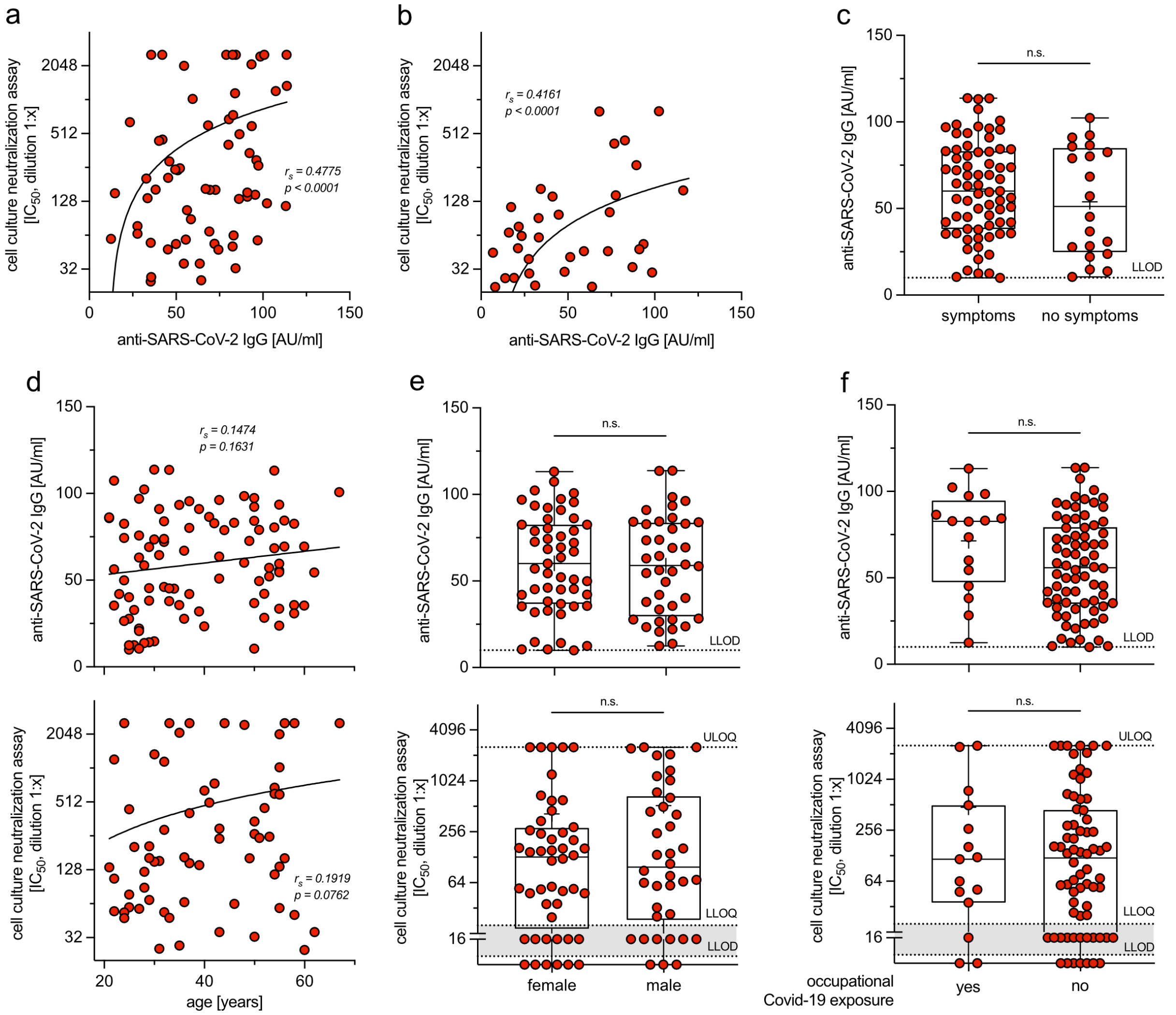

Supplementary Figure 2 | Characteristics of the dynamics of anti-SARS-CoV-2 IgG levels and virus-neutralization activities in convalescent individuals

(a,b) correlation of anti-SARS-CoV-2 IgG levels with virus-neutralization activity of convalescents at month 2 (a) and month 5 (b) after SARS-CoV-2 infection, 21 samples (a) or 57 samples (b) were below the quantification limit and are not shown but were included in the statistical analysis. (c) reporting of symptoms (n=71) or no symptoms (n = 20) in convalescents and anti-SARS-CoV-2 IgG levels. LLOD – lower limit of detection. Data is shown, respectively, as median (60.13 and 51.18) with box bounds at 25% (38.15 and 24.74) and 75% percentile (83.09 and 84.95), whiskers show maxima (113.8 and 102.3) and minima (10.01 and 10.52). (d) correlation of anti-SARS-CoV-2 IgG levels and virus-neutralization activity with age of convalescents. 21 samples were below the quantification limit and are not shown (lower row). (e,f) anti-SARS-CoV-2 IgG levels and virus-neutralization activity in convalescents according to sex (n = 51, n = 48 (female); n = 40, n = 38 (male), respectively) or occupational exposure to COVID-19 patients (n = 16, n = 15 (yes); n = 74, n = 70 (no), respectively). LLOQ – lower limit of quantification; ULOQ – upper limit of quantification. Data is shown, respectively, as median (f: 60.13, 129.00 and m: 59.01, 97.75; yes: 82.73, 116.6 and no: 55.93, 120.8) with box bounds at 25% (f: 36.74, 18.23 and m: 29.61, 23.13; yes: 47.55, 35.80 and no: 34.91, 16.00) and 75% percentile (f: 82.56, 284.00 and m: 83.75, 672.70; yes: 94.71, 505.7 and no: 79.33, 444.8), whiskers show maxima (f: 113.2, 2560 and m: 113.8, 2560; yes: 113.2, 2560 and no: 113.8, 2560) and minima (f: 10.01, 1.0 and m: 12.51, 1.0; yes: 12.45, 1.0 and no: 10.01, 1.0). Statistical analyses by two-sided Mann-Whitney (c,e,f), and by Spearman correlation (a,b,d); r_s denotes Spearman correlation coefficient; n.s. denotes not significant.

a

	CV (%)			
Parameter	IFNγ	IL-2	TNF	GzmB
Intra-assay variation (well to well; 4 replicates)	4.9	6.6	5.5	13.6
Inter-assay variation (plate to plate)	3.1	2.7	5.0	8.1
Inter-assay variation (day to day)	5.5	8.4	8.4	10.8
Inter-operator	6.6	11.1	4.8	6.7

b

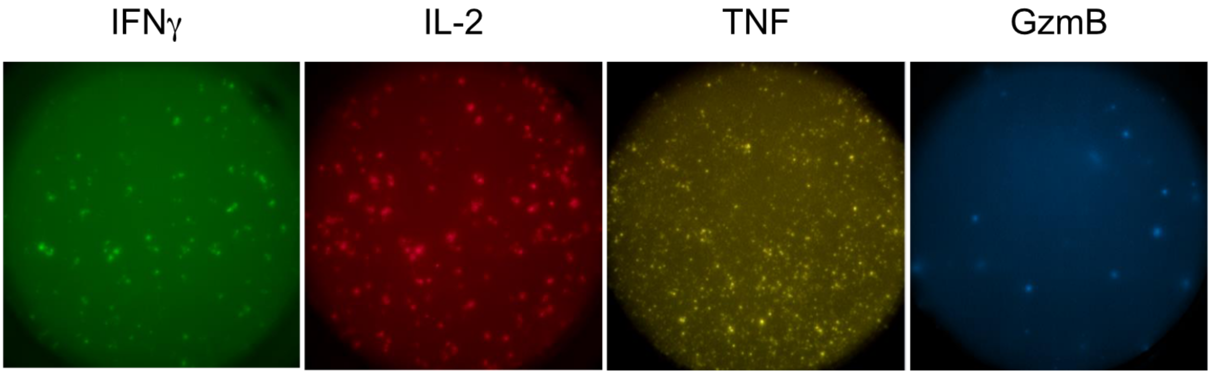

Supplementary Figure 3 | Performance of the four-color Fluorospot assay to detect spike-reactive cytokine-secreting cells directly *ex vivo* in 10^6 peripheral blood mononuclear cells (PBMCs)

(a) report of the Fluorospot assay characteristics. (b) representative images of the Fluorospot assay detecting frequencies of cytokine secreting cells among 10^6 PBMCs from a convalescent individual stimulated with the S1-peptide pool.

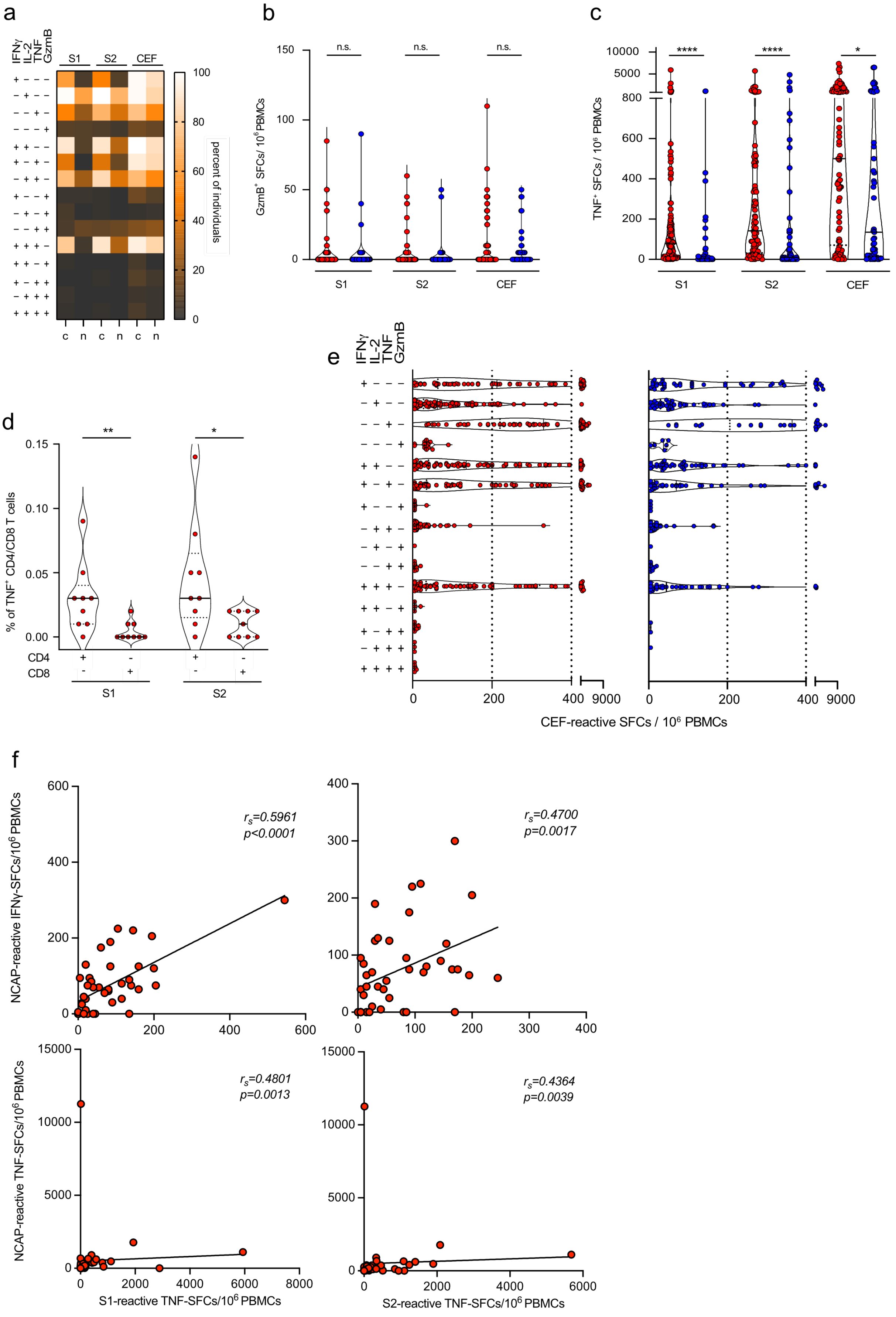
a
IFNγ
IL-2
TNF
GzmB
S1
S2
CEF
c n c n c n
percent of individuals
b
GzmB+ SFCs/ 10⁶ PBMCs
n.s.
S1
S2
CEF
c
TNF+ SFCs / 10⁶ PBMCs
****
*
d
% of TNF+ CD4/CD8 T cells
**
CD4
CD8
e
CEF-reactive SFCs / 10⁶ PBMCs
f
NCAP-reactive IFNγ-SFCs/10⁶ PBMCs
rs=0.5961
p<0.0001
rs=0.4700
p=0.0017
NCAP-reactive TNF-SFCs/10⁶ PBMCs
rs=0.4801
p=0.0013
rs=0.4364
p=0.0039
S1-reactive TNF-SFCs/10⁶ PBMCs
S2-reactive TNF-SFCs/10⁶ PBMCs

Supplementary Figure 4 | Characteristics of spike-reactive and CEF-reactive cytokine-secreting cells in convalescents and naïve individuals

(a) heatmap of individuals with mono- or polyfunctional S1-/S2-/CEF-reactive cytokine-secreting cells in convalescents (c) and naïve individuals (n) by direct *ex vivo* Fluorospot analysis at month 5 after SARS-CoV-2 infection**.** (b) frequencies of S1-/S2-/CEF-reactive GzmB-secreting cells; convalescents (red, n = 88), naïve individuals (blue, n = 52). (c) S1-/S2-/CEF-reactive TNF-secreting cells by direct *ex vivo* Fluorospot analysis; convalescents (red, n = 88), naïve individuals (blue, n = 52). (d) frequencies of S1- or S2-reactive TNF-secreting CD4 or CD8 T cells in convalescents (red, n = 9) determined by direct *ex vivo* intracellular cytokine staining and flow cytometric detection. (e) frequencies of mono- and poly-functional CEF-reactive cytokine-secreting cells by direct *ex vivo* Fluorospot analysis. (f) correlation of the frequencies of nucleocapsid- (NCAP) and S1- and S2-reactive T cells determined by Fluorospot analysis in convalescents at month 5; convalescents (red), naïve individuals (blue). Statistical analyses by two-sided Mann-Whitney, two-sided Wilcoxon signed-rank tests and by Spearman correlation and linear regression (b,c,d,f); rs denotes Spearman correlation coefficient; n.s. denotes not significant; *p<0.05; **p<0.01; ****p< 0.0001.

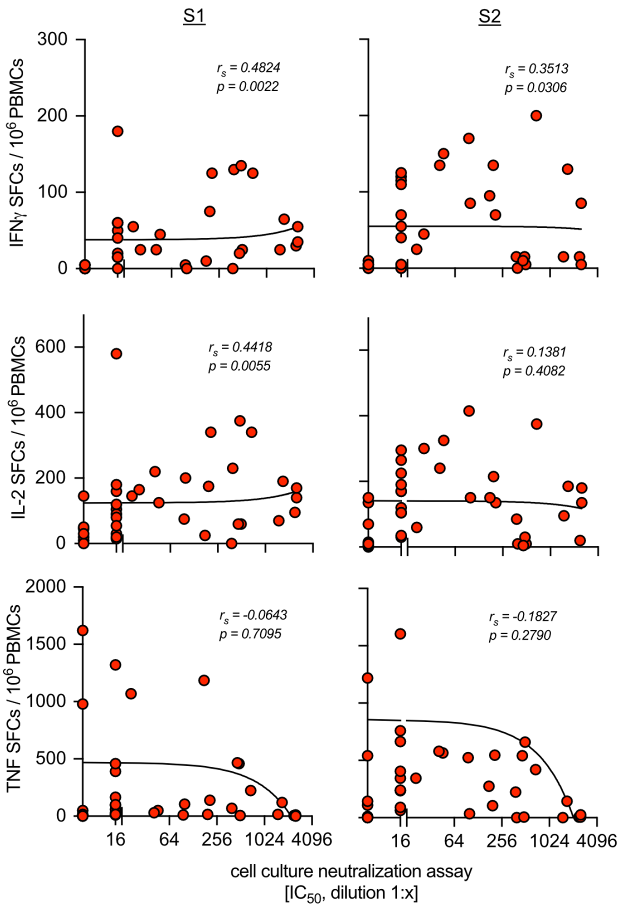

Supplementary Figure 5 | Correlation of spike-reactive cells with spike-specific antibodies and neutralization activity and dynamics of spike- and CEF-reactive cytokine-secreting cells over time

Spearman correlation of S1- and S2-reactive cytokine-secreting cells from convalescents with cell culture virus-neutralization activity of serum at month 11 after SARS-CoV-2 infection. r_s denotes Spearman correlation coefficient.

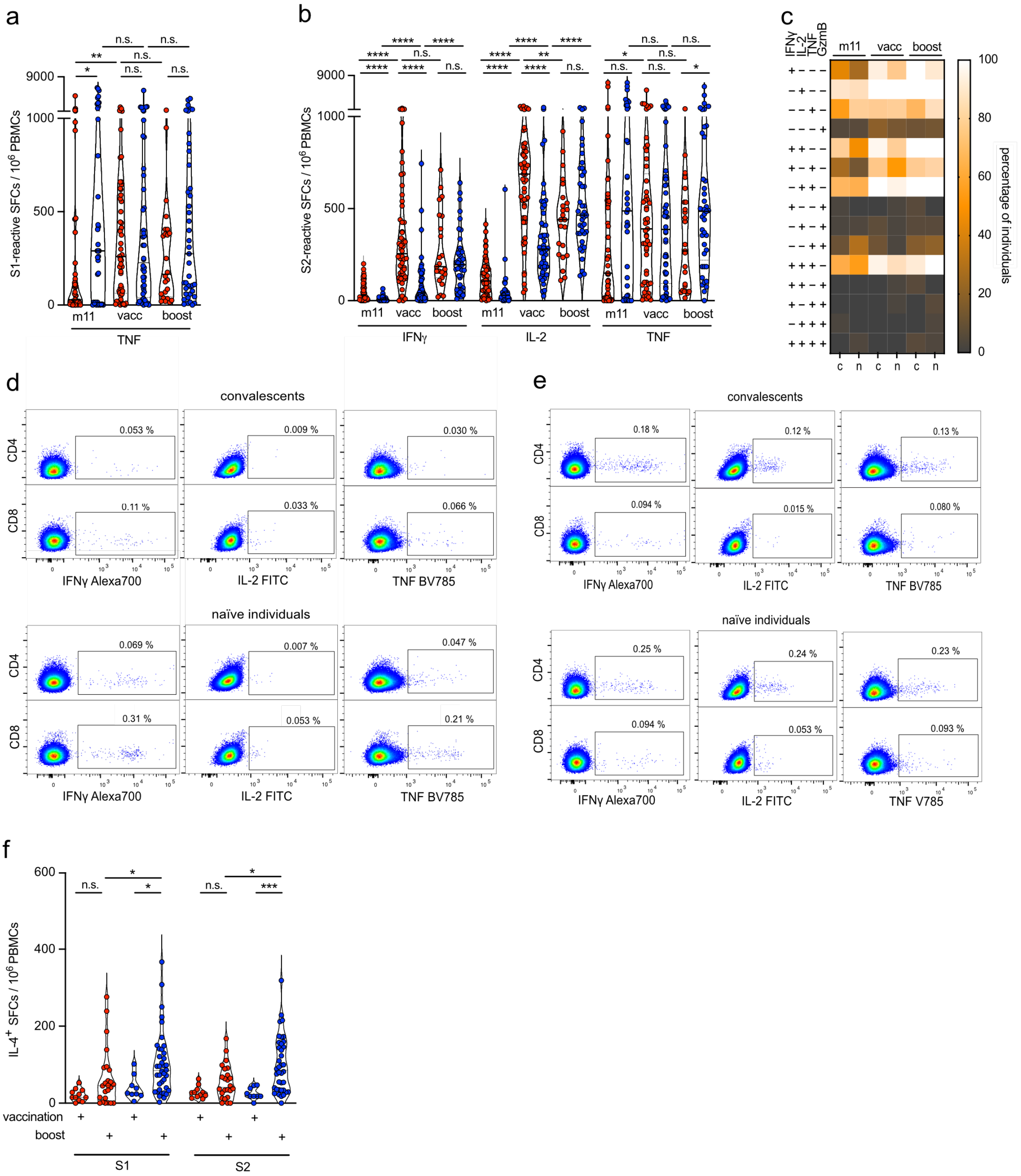

Supplementary Figure 6 | Frequencies of spike-reactive cytokine-secreting cells at month 11 after SARS-CoV-2 infection or after BNT162b2 mRNA prime and boost vaccination

(a,b) frequencies of spike-reactive cytokine-secreting cells determined directly *ex vivo* by Fluorospot analysis at month 11 after SARS-CoV-2 infection, at 2 weeks after BNT162b2 mRNA prime vaccination (vacc) or at 2 weeks after boost vaccination (boost); convalescents (red, n=50 (m11), n=54 (vacc), n=23 (boost)), naïve individuals (blue, n = 39 (m11), n = 49 (vacc), n = 40 (boost)). (c) heatmap of frequencies of individuals bearing spike-reactive cytokine-secreting cells, c - convalescents, n - naïve individual. (d,e) intracellular cytokine staining of spike-reactive CD4 and CD8 T cells determined directly *ex vivo* by flow cytometry, time points as in (a). (f) frequencies of spike-reactive IL-4-secreting cells after BNT162b2 mRNA prime vaccination (vacc) or boost vaccination (boost); convalescents (red, n = 11 (vacc), n = 24 (boost)), naïve individuals (blue, n = 9 (vacc), n = 38 (boost)). Statistical analyses by ANOVA, two-sided Mann-Whitney and two-sided Wilcoxon signed-rank tests (a, b, f); n.s. denotes not significant, *p<0.05; **p<0.01; ***p< 0.001; ****p< 0.0001.

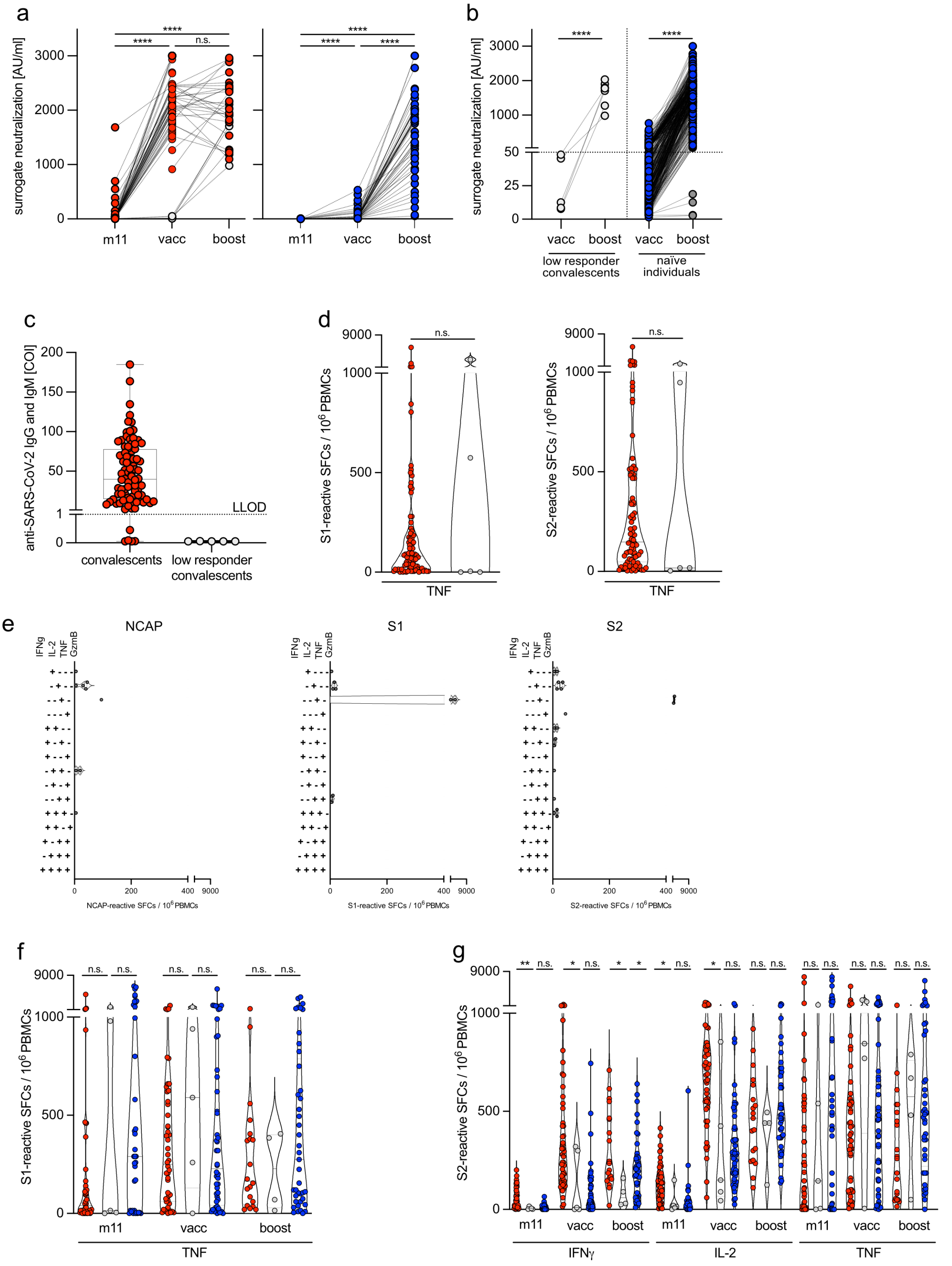

Supplementary Figure 7 | Lack of spike-specific immunity in convalescents with a delayed response to BNT162b2 mRNA vaccination

(**a**) individual courses of surrogate virus-neutralization activity in convalescents (red, n = 59 (m11), n = 82 (vacc), n = 33 (boost)) after prime and boost BNT162b2 mRNA vaccination compared to naïve individuals (blue, n=36 (m11), n = 51 (vacc), n = 49 (boost)). (**b**) individual courses of surrogate virus-neutralization activity in a separate cohort of naïve individuals (n = 455) after prime and boost BNT162b2 mRNA vaccination (right panel) demonstrating four individuals with a low response to vaccination (dark grey), compared to virus-neutralization

activity in low responder convalescents (n = 5) after vaccination (left panel). (**c**) anti-SARS-CoV2 IgG levels in low-level non-responder convalescents (grey, n = 5) compared to convalescents (red, n = 86) at month 2. (d) spike-reactive TNF-secreting cells at month 5 after infection in convalescents (red, n = 83) and convalescents with low response to vaccination (grey, n = 5). (e) fluorospot analysis revealing the frequencies of spike-reactive and nucleocapsid-reactive mono- and polyfunctional cytokine-secreting cells (spot-forming cells/SFCs in 10^6 PBMCs from low responder convalescents at month 5 after SARS-CoV-2 infection. (f,g) frequencies of spike-reactive cytokine-secreting cells at month 11 after SARS-CoV-2 infection, two weeks after prime (vacc) and two weeks after boost vaccination (boost) in convalescents (red, n = 45 (m11), n = 49 (vacc), n = 19 (boost)), convalescents with low response to vaccination (grey, n = 5 at all time points) and naive individuals (blue, n = 39 (m11), n = 49 (vacc), n=40 (boost)). Statistical analyses by ANOVA, two-sided Mann-Whitney and two-sided Wilcoxon signed-rank tests (a,b,c,d,f,g); n.s. denotes not significant; * $p<0.05$; ** $p<0.01$; **** $p< 0.0001$

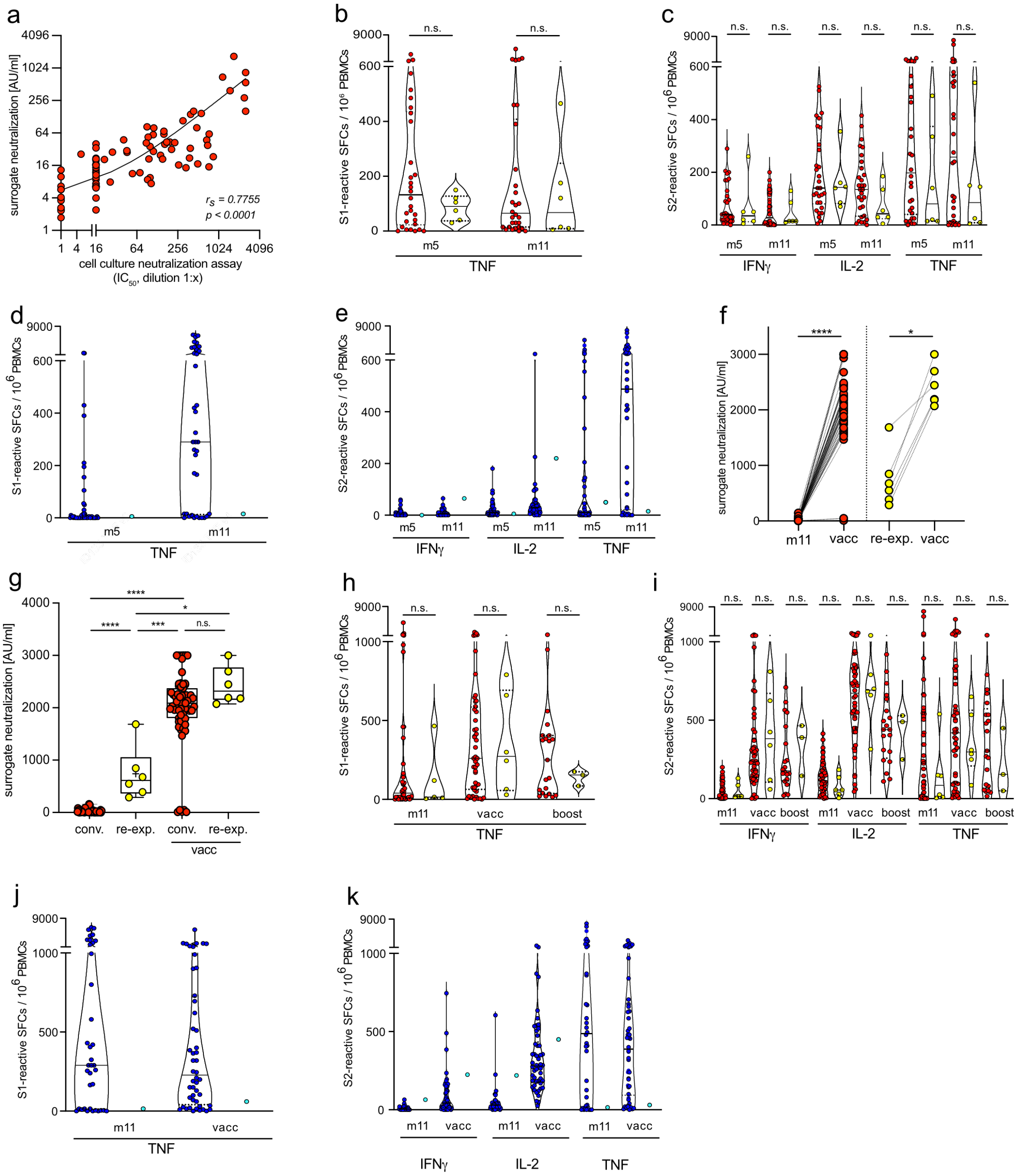

Supplementary Figure 8 | Characteristics of spike-specific immunity in convalescents compared to an individual after SARS-CoV-2 infection

(a) correlation between cell culture neutralization assay (shown as dilution 1:x mediating a 50% inhibition of SARS-CoV-2 infection *in vitro* - IC50) with a competitive CLIA (YHLO) measuring surrogate neutralization (arbitrary units (AU)/ml) at month 5 after SARS-CoV-2 infection. (b,c) frequencies of S1- or S2-reactive cytokine-secreting cells in convalescents (red, n = 32 (m5), n = 30 (m11)) or convalescents with increased virus-neutralizing activity (yellow, n = 6) at month 5 and month 11 after SARS-CoV-2 infection. (d,e) frequencies of S1- or S2-reactive cytokine-secreting cells in naïve individuals (blue, n = 51 (m5), n=39 (m11)) or the infected individual (turquoise, n = 1) at month 5 and month 11 after SARS-CoV-2 infection. (f) individual analysis of surrogate neutralization activity before and after BNT162b2 mRNA vaccination in convalescents (red, n = 60 (m11), n = 63 (vacc)) or convalescents with increased virus-neutralizing activity (yellow, n = 6). (g) surrogate neutralization activity in convalescents (red, n = 60 (m11), n = 63 (vacc)) and convalescents with increased virus-neutralizing activity (yellow, n = 6) before and after BNT162b2 mRNA vaccination. Data is shown, respectively, as median

(m11: 16.81, 613.2 and vacc: 2087, 2319) with box bounds at 25% (m11:7.275, 363.10 and vacc: 1801, 2153) and 75% percentile (m11: 33.15, 1055 and vacc: 2376, 2772), whiskers show maxima (m11: 161.9, 1684 and vacc: both 3000) and minima (m11: 1.73, 286.5 and vacc: 7.73, 2072). (h,i) frequencies of S1 or S2-reactive cytokine-secreting cells in convalescents (red, n = 44 (month 11), n = 48 (vacc), n = 20 (boost)) or convalescents with increased virus-neutralizing activity (yellow, n = 6) before vaccination (month 11), 2 weeks after vaccination (vacc) and 2 weeks after boost vaccination (boost). (j,k) frequencies of S1- or S2-reactive cytokine-secreting cells in naïve individuals (blue, n = 39 (month 11), n = 49 (vacc), n = 40 (boost)) or the infected individual (turquoise, n = 1) before vaccination (month 11) and 2 weeks after vaccination (vacc). Statistical analyses by ANOVA, two-sided Mann-Whitney, two-sided Wilcoxon signed-rank tests (b,c,f,g,h) and by Spearman correlation (a) and linear regression. r_s denotes Spearman correlation coefficient; n.s. denotes not significant *$p<0.05$; ***$p< 0.001$; ****$p< 0.0001$

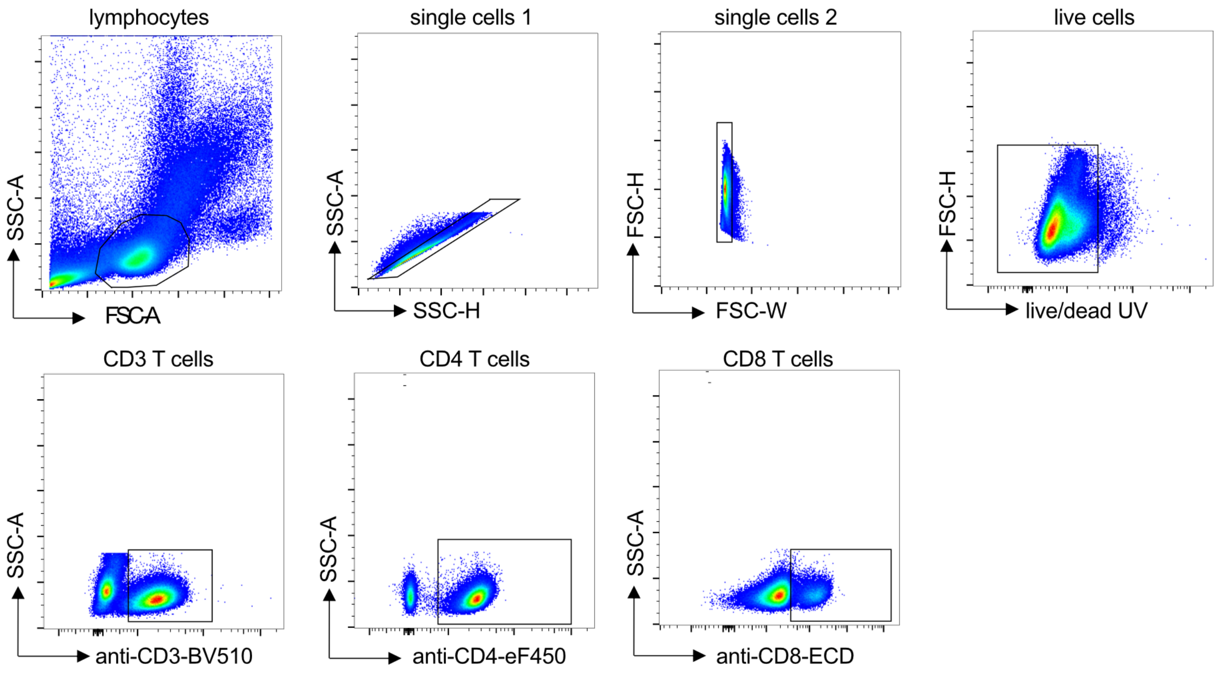

Supplementary Figure 9 | Gating strategy for flow cytometry.

Peripheral blood lymphocytes were gated based on size, single cells, live/dead staining, staining with anti-CD3 to identify T cells. Subsequent differentiation of T cell populations was based on CD4 and CD8 surface staining.

Data availability: All data generated in this study are provided in the Supplementary Information/Source Data Files. The study protocols for the SeCoMRI and VaCoMRI studies are available upon request.
