# Supplementary material for: Dynamics of spike-and nucleocapsid specific immunity during long-term follow-up and vaccination of SARS-CoV-2 convalescents
Source: Nat Commun. 2022 Jan 10;13:153. doi: 10.1038/s41467-021-27649-y (PMC8748966; doi:10.1038/s41467-021-27649-y)
Supplement: Supplementary file 3 — Reporting Summary [file 41467_2021_27649_MOESM3_ESM.pdf]

nature portfolio

Corresponding author(s): Percy A Knolle

Last updated by author(s): 11/18/2021

Reporting Summary

Nature Portfolio wishes to improve the reproducibility of the work that we publish. This form provides structure for consistency and transparency in reporting. For further information on Nature Portfolio policies, see our Editorial Policies and the Editorial Policy Checklist.

Statistics

For all statistical analyses, confirm that the following items are present in the figure legend, table legend, main text, or Methods section.

n/a	Confirmed	
☐	☒	The exact sample size (*n*) for each experimental group/condition, given as a discrete number and unit of measurement
☐	☒	A statement on whether measurements were taken from distinct samples or whether the same sample was measured repeatedly
☐	☒	The statistical test(s) used AND whether they are one- or two-sided<br>*Only common tests should be described solely by name; describe more complex techniques in the Methods section.*
☐	☒	A description of all covariates tested
☐	☒	A description of any assumptions or corrections, such as tests of normality and adjustment for multiple comparisons
☐	☒	A full description of the statistical parameters including central tendency (e.g. means) or other basic estimates (e.g. regression coefficient) AND variation (e.g. standard deviation) or associated estimates of uncertainty (e.g. confidence intervals)
☒	☐	For null hypothesis testing, the test statistic (e.g. F, t, r) with confidence intervals, effect sizes, degrees of freedom and P value noted<br>*Give P values as exact values whenever suitable.*
☒	☐	For Bayesian analysis, information on the choice of priors and Markov chain Monte Carlo settings
☒	☐	For hierarchical and complex designs, identification of the appropriate level for tests and full reporting of outcomes
☒	☐	Estimates of effect sizes (e.g. Cohen's d, Pearson's r), indicating how they were calculated

Our web collection on statistics for biologists contains articles on many of the points above.

Software and code

Policy information about availability of computer code

Data collection	all data from participants were only obtained after informed written consent
Data analysis	data from Fluorospot analysis, flow cytometry, cell-based infection inhibition assays and serological assays for detection of SARS-CoV-2 specific antibodies were analysed with Prism software (vs9), flow jow software was used to analyse flow cytometric data; ImmunoSpot 7.0.17.0 Professional DC Software

For manuscripts utilizing custom algorithms or software that are central to the research but not yet described in published literature, software must be made available to editors and reviewers. We strongly encourage code deposition in a community repository (e.g. GitHub). See the Nature Portfolio guidelines for submitting code & software for further information.

Data

Policy information about availability of data

All manuscripts must include a data availability statement. This statement should provide the following information, where applicable:

- Accession codes, unique identifiers, or web links for publicly available datasets
- A description of any restrictions on data availability
- For clinical datasets or third party data, please ensure that the statement adheres to our policy

all data are provided as source data with the submitted manuscript, further more detailed raw data are available upon request

Field-specific reporting

Please select the one below that is the best fit for your research. If you are not sure, read the appropriate sections before making your selection.

☒ Life sciences ☐ Behavioural & social sciences ☐ Ecological, evolutionary & environmental sciences

For a reference copy of the document with all sections, see nature.com/documents/nr-reporting-summary-flat.pdf

Life sciences study design

All studies must disclose on these points even when the disclosure is negative.

Sample size	We included all individuals who gave written informed consent to participate in the study. The number of tested individuals and the numbers of seropositive individuals are given in the detailed description. Only individuals with more than two independent positive tsts for anti-SARS-CoV-2 IgG were included as seropositive individuals.
Data exclusions	Dead cells were excluded from further analysis in flow cytometry. Only individuals with more than two independent tests for anti-SARS-CoV-2 IgG were included as seropositive individuals.
Replication	All assays conducted have been thoroughly evaluated for their performance and their high reproducibility (details given in Extended Data Fig. 3). Given the scarcity of the clinical material and the necessity to work with freshly isolated PMBCs of study participants no technical replicates were performed for Fluorospot analysis and flow cytometry analysis. As we have followed individuals over time, repeated measurement from different time points are available for the same persons. Infection neutralization experiments were performed in duplicates. CE-IVD-certified diagnostic assays were performed without technical replicates.
Randomization	The control cohort of seronegative individuals was matched according to age, sex, working conditions, risk factors etc with the cohort of seropositive individuals.
Blinding	all experimental procedures measurement and all analyses were done in a blinded fashion and only the final analysis of the results and preparation of figures was performed by separate persons.

Reporting for specific materials, systems and methods

We require information from authors about some types of materials, experimental systems and methods used in many studies. Here, indicate whether each material, system or method listed is relevant to your study. If you are not sure if a list item applies to your research, read the appropriate section before selecting a response.

Materials & experimental systems

n/a	Involved in the study	
☐	☒	Antibodies
☐	☒	Eukaryotic cell lines
☒	☐	Palaeontology and archaeology
☒	☐	Animals and other organisms
☐	☒	Human research participants
☒	☐	Clinical data
☒	☐	Dual use research of concern

Methods

n/a	Involved in the study	
☒	☐	ChIP-seq
☐	☒	Flow cytometry
☒	☐	MRI-based neuroimaging

Antibodies

Antibodies used

Human IFN-γ/IL-2/TNF-α/Granzyme B Four-Color FluoroSpot kit (Lots I2TZ0203JEWF, I2TZ0520JEWF, I2TZ0929JEWF, I2TZ0114KEWF) including following antibodies: Human IFN-γ Capture Ab, Human IL-2 Capture Ab, Human TNF-α Capture Ab, Human GrzB Capture Ab; anti-human IFNgamma (FITC), anti-human IL-2 (Hapten2), anti-human TNF (Hapten1), anti-human Granzyme B (Biotin) and anti-FITC Alexa Fluor® 488 (visualizes IFNgamma), anti-Hapten2 CTL-Red™ (visualizes IL-2), anti-Hapten1 CTLYellow™ (visualizes TNF), and Streptavidin eFluor® 450 (visualizes GzmB)
Human IL-5/IL-4 Double-Color FluoroSpot (Lots 540925JEWF and 540112KEWF) including following antibodies: Human IL-5 Capture Ab, Human IL-4 Capture Ab, anti-human IL-5 (Hapten3), anti-human IL-4 (Biotin) and anti-Hapten3 Alexa Fluor® 488 (visualizes IL-5) and Strep CTL-Red™ (visualizes IL-4).

Antibodies used in flow cytometry
Purified mouse anti-human CD28 BD Biosciences n.a. 1.0 Cat# 555725 0301950
BV510 mouse anti human CD3 BioLegend SK7 1.0 Cat# 344828 B310747
EF450 mouse anti human CD4 eBiosciences SK3 0.5 Cat# 48-0047-42 2011201
ECD mouse anti human CD8 Beckman Coulter SFCI21Thy2D3 0.2 Cat# 6604728 7974074
Al700 mouse anti human IFN-γ BD Biosciences B27 0.1 Cat# 557995 7159617

	BV785 mouse anti human TNF-a BioLegend MAb11 5.0 Cat# 502948 B311979<br>FITC rat anti human IL-2 eBiosciences MQ1-17H12 3.1 Cat# 11-7029-42 2153333
Validation	all antibodies used were commercially available and have been tested for specificity according to the suppliers reagent-specific information

Eukaryotic cell lines

Policy information about cell lines

Cell line source(s)	Vero cells
Authentication	obtained from verified source (ATCC)
Mycoplasma contamination	excluded by PCR testing
Commonly misidentified lines<br>(See ICLAC register)	*Name any commonly misidentified cell lines used in the study and provide a rationale for their use.*

Human research participants

Policy information about studies involving human research participants

Population characteristics	The initial cohort for detection of seropositive individuals comprized a total of 108 seropositive convalescents and 4446 naive individuals. Confirmation of previous SARS-CoV-2 infection (≥2 independent serological test results using CE-IVD-certified tests) identified 94 convalescents, from whom 91 agreed to participate in the study. This group of convalescents consisted of 51 female (56%) and 40 male (44%) convalescents, compared to a control cohort of naive individuals comprizing 33 female (62,3%) and 20 male (37.7%) individuals. Mean age of the convalescent individuals was 39 ±13 years compared to a mean age of 42±20 years of the naive individuals. 49 (54%) of convalescents reported patient contact w, compared to 27 (50.9%) of naive individuals. 16 (17.6%) of convalescents reported contact with COVID-19 patients, compared to 14 /26.4%) of naive individuals. Risk factors (smoking) were less prevalent in convalescents 7 (7.7%) than in naive individuals (11 (20.8%). No differences existed with respect to pre-existing conditions, in particular lung disease, cardiovascular disease and diabetes.
Recruitment	All health care workers at the University Hospital München were invited to participate in the study. They received information by e-mail, handout and information material distributed through official communication channels of the University hospital. While we attempted to avoid any recruitment bias, we cannot exclude that particular groups of health care workers may have received less information with an unknown effect on the recruitment process.
Ethics oversight	The study was approved by the local ethics committee of the Technical University of Munich.

Note that full information on the approval of the study protocol must also be provided in the manuscript.

Flow Cytometry

Plots

Confirm that:

- [x] The axis labels state the marker and fluorochrome used (e.g. CD4-FITC).
- [x] The axis scales are clearly visible. Include numbers along axes only for bottom left plot of group (a 'group' is an analysis of identical markers).
- [x] All plots are contour plots with outliers or pseudocolor plots.
- [x] A numerical value for number of cells or percentage (with statistics) is provided.

Methodology

Sample preparation	Sample preparation (Describe sample preparation)<br>1 x 106 freshly isolated PBMCs were transferred into 150 µL RPMI1640 medium supplemented with 10% FCS and 1% penicillin-streptomycin (PenStrep, Life Technologies, Invitrogen, Germany) (abbr.: RPMI-10) containing costimulatory antibodies to ensure effective T cell stimulation (1 µg/mL anti-CD28; BD Biosciences, Germany) in one well of a 96-well polypropylene U-bottom microtiter plate. Cells were stimulated with the S1/S2 peptide pools (1 µg/mL). After one hour of incubation at 37°C in 5% CO2, 10 µg/mL of Brefeldin A (Sigma-Aldrich, Germany) was added to the cell suspension and incubated for 4h at 37°C in 5% CO2. For intracellular cytokine staining, PBMCs were labelled with the LIVE/DEAD™ Fixable Blue Dead Cell Stain Kit (Thermo Fisher Scientific, USA) in a total volume of 100 µL for 30 min at 4°C in the dark, and washed twice with 200 µL FACS buffer (BD Biosciences). After centrifugation (560g, 4°C, 5 min), PBMCs were fixed for 20 min at 4°C in the dark in 100 µL of an intracellular fixation buffer (Intracellular Fixation Buffer, Thermo Fisher Scientific, USA). After two wash steps with 200 µL/well Perm/Wash solution (Cytofix/Cytoperm Kit; BD Biosciences) and a centrifugation step (710g, 4°C, 5 min), PBMCs were stained with the antibodies listed listed below in a total volume of 80 µL Perm/Wash buffer including a brilliant violet buffer (BD Pharmingen Stain Buffer, BD Biosciences) for 30 min at 4°C in the dark. Single color compensation was performed using 25 µL of compensation beads (UltraComp eBeads and ArC™ Amine Reactive Compensation Bead Kit for LIVE/DEAD compensation, both from Thermo Fisher Scientific, USA) following the instructions of the manufacturer. Cells and beads were washed twice and finally re-suspended in 300 µL FACS buffer for acquisition. Cells were stored cold and in the

dark until acquisition.

Fluorospot
Sample preparation
PBMCs were used directly after isolation and placed at 2 × 105 (IFN/IL-2/TNF/GzmB Fluorospot; 4CFS) or 8 × 105 /well (IL-4/IL-5 Fluorospot; 2CFS) in a final volume of 200 µL/well. PBMCs were then stimulated for 22h (4CFS) or 48h (2CFS) with 1 µg/mL of overlapping peptide pools (15mers overlapping by 11 aa) of the SARS-CoV-2 spike protein (PepMix™ SARS-CoV-2 (PM-WCPV-S), consisting of two peptide pools, i.e. S1 and S2 with 158 and 157 peptides, respectively) (JPT Peptide Technologies, Germany). After the stimulation period, the plates were washed and 80 µL of either anti-human IFN (FITC), anti-human IL-2 (Hapten2), anti-human TNF (Hapten1), and anti-human Granzyme B (Biotin) or anti-human IL-5 (Hapten3) and anti-human IL-4 (Biotin) detection antibody solution was added for additionally 2h. For the visualization of secreted cytokines and GzmB, plates were washed and a tertiary solution including either anti-FITC Alexa Fluor® 488 (visualizes IFN), anti-Hapten2 CTL-Red™ (visualizes IL-2), anti-Hapten1 CTLYellow™ (visualizes TNF), and Streptavidin eFluor® 450 (visualizes GzmB) or anti-Hapten3 Alexa Fluor® 488 (visualizes IL-5) and Strep CTL-Red™ (visualizes IL-4) was added for one hour. The staining procedure was stopped by washing the plate.

Instrument	BD Fortessa Cell Analyzer and ImmunoSpot® Series 6 Ultimate UV Image Analyzer
Software	FACS DIVA Software
Cell population abundance	> 10E5 Cells per sample
Gating strategy	Gating strategy for flow cytometric analysis of ex vivo re-stimulated PBMCs is shown in the supplementary information (supplementary data I). Cells were gated on size (SSC-A vs. FSC-A), single cells (SSC-A vs. SSC-H and FSC-H vs. FSC-W), live cells (FSC-H vs. Live/Dead UV), and CD3 positive cells (FSC-H vs. CD3 BV510) to identify T cells. Subsequent division of T cell subsets was based on CD4 (SSC-A vs. CD4 eF450) and CD8 (SSC-A vs. CD8 ECD) surface staining. Subsequently, cytokine expressing cells were gated in the CD4 and CD8 populations (SSC vs. IFN- Al700; SSC vs. IL-2 FITC and SSC vs. TNF BV785), respectively.

☒ Tick this box to confirm that a figure exemplifying the gating strategy is provided in the Supplementary Information.
